# Whole-lesion ADC histogram analysis is not able to reflect microvessel density in HNSCC

**DOI:** 10.1097/MD.0000000000015520

**Published:** 2019-05-24

**Authors:** Hans-Jonas Meyer, Gordian Hamerla, Leonard Leifels, Anne Kathrin Höhn, Alexey Surov

**Affiliations:** a Department of Diagnostic and Interventional Radiology; b Department of Pathology, University of Leipzig, Leipzig, Germany.

**Keywords:** ADC, histogram analysis, HNSCC, MVD

## Abstract

Diffusion-weighted imaging (DWI) is a functional imaging technique sensitive to microstructure in tissues. It is widely acknowledged to reflect cellularity in tumors. A small part of DWI is also sensitive to perfusion-related information and might therefore be also be able to reflect microvessel density in tumor tissues. Aim of the present study was to elucidate possible correlations between microvessel density and apparent diffusion coefficient (ADC) values in head and neck squamous cell carcinoma (HNSCC).

Thirty-four patients with histologically proven primary HNSCC were included in the study. DWI was performed with a 3 T magnetic resonance imaging (MRI) (b-values 0 and 800 s/mm^2^) and histogram analysis was calculated with a whole lesion measurement. In every case, microvessel density was estimated with CD105-stained specimens.

There were no statistically significant correlations between ADC histogram parameters and microvessel density. The calculated correlation coefficients ranged from *r* = -0.27, *P* = .13 for entropy and vessel area to *r* = 0.16, *P* = .40 for ADCmin and vessel count.

Whole-lesion histogram analysis of ADC values cannot reflect microvessel density in HNSCC.

## 1 Introduction

Head and neck squamous cell carcinoma (HNSCC) is one of the most frequent malignancies.^[1]^ The role of imaging modalities is to locate the tumor, detect infiltration of adjacent structures, and to rule out possible metastasis.^[2]^ However, modern functional imaging modalities, such as diffusion-weighted imaging (DWI), quantified by apparent diffusion coefficients (ADCs) or dynamic contrast-enhanced MRI (DCE-MRI), can provide additional information regarding tumor microstructure, such as cellularity and microvessel density (MVD).^[3–7]^ According to the literature, DWI and DCE-MRI can reflect several different histopathological features and predict treatment success in different tumors in oncology.^[3–7]^ However, the associations between imaging modalities and histopathology parameters seem to significantly differ between tumor entities.^[6,8,9]^

DWI is usually acquired by 2 b-values, a low one, usually 0 s/mm^2^ and a high one, usually 800 to 1000 s/mm^2^.^[10]^ It is known that the low signal intensity of DWI, up to 200 s/mm^2^, is more sensitive to perfusion than diffusion and might, therefore, be able to also reflect perfusion-related parameters in tissues, such as vessel density.^[10,11]^ This might be of crucial importance because MVD has been shown to be an important prognostic factor in various tumors, including HNSCC.^[12–14]^

Nowadays, an emergent imaging analysis, namely histogram analysis, is used to further analyze radiological images. With this approach, every voxel of a regions of interest (ROI) is issued into a histogram and, therefore, investigated tumors can be better characterized.^[15]^ Presumably, heterogeneity of the histogram might display heterogeneity of the tumor. The resulting parameters are as follows: minimum, mean, maximum, median, mode, several percentiles, and second order statistics, namely kurtosis, skewness, and entropy.^[15]^ Using this technique as a whole-lesion measurement, tumor microstructure might be better visualized and also be more sensitive to reflect perfusion aspects in HNSCC.

There is ongoing debate, whether a clinically used DWI-MRI protocol can reflect MVD in tumors due to the above-mentioned possible perfusion-related aspects of DWI. Thereby, DWI might not only be capable to assess cellularity in tissues but also microvessel-related features of tumors, which might provide crucial information for clinical routine. For example, it was shown that ADC values might be associated with expression of vascular endothelial growth factor (VEGF) in several tumor entities, including prostate cancer,^[16]^ thyroid nodules,^[17]^ and rectal cancer.^[18]^ VEGF is the most important factor to induce angiogenesis.^[19]^ When ADC values are linked to VEGF expression, they might also be associated with MVD.

This might establish the opportunity for DWI to display treatment response to anti-angiogenesis therapy, which is increasingly used in various tumor treatment regimes.

To date, no study investigated possible correlations between MVD and ADC values in HNSCC. Therefore, we conducted the present study to elucidate possible associations between MVD and histogram parameters derived from ADC maps in HNSCC.

## 2 Materials and methods

This retrospective study was approved by the institutional review board (Ethic committee of the University of Leipzig, study codes 180–2007, 201–10–12072010, and 341–15–05102015). All methods were performed in accordance with the relevant guidelines and regulations. All patients gave their written informed consent.

### 2.1 Patients

Overall, 34 patients with primary HNSCC of different localizations were enrolled in the present study. There were 9 (26%) women and 25 (74%) men with a mean age of 56.7 ± 10.2 years, range 33 to 77 years.

### 2.2 DWI

DWI was obtained by using of an axial EPI (echo-planar imaging) sequence (TR/TE 8620/73 ms, slice thickness 4 mm, voxel size 3.2 × 2.6 × 4.0 mm) with b-values of 0 and 800 s/mm^2^. ADC maps were saved in DICOM format and processed offline with custom-made Matlab-based application (The Mathworks, Natick, MA). Polygonal ROI were manually drawn on the transferred ADC maps along the contours of the primary tumor on each slice (whole lesion measurement) according to the contrast-enhanced t1-weighted images. All measures were performed by one radiologist (A.S., 15 years radiological experience). The following parameters were calculated: mean ADC (ADC_mean_), maximum ADC (ADC_max_), minimum ADC (ADC_min_), median ADC (ADC_median_), mode ADC (ADC_mode_). Furthermore, ADC percentiles were 10th (P10 ADC), 25th (P25 ADC), 75th (P75 ADC), and 90th (P90 ADC), kurtosis, skewness, and entropy.

### 2.3 Histopathological analysis

In all cases, the diagnosis of HNSCC was confirmed histopathologically by biopsy. Representative tumor tissue slides from formalin-fixed paraffin-embedded tissue were processed after deparaffinization. The specimens were stained with CD 105 antigen. All stained samples were digitalized by using a research microscope Jenalumar (Zeiss, Jena, Germany), with camera diagnostic instruments 4.2., magnification x400. Furthermore, the digital histopathological images were transferred as uncompressed TIFF images to ImageJ software (version 1.48 v; NIH, Bethesda, MD). MVD included the following parameters: stained vessel area (% per high power field), calculated as CD 105 positive area divided by the total area of the analyzed histological specimens and total number of vessels according to Weidner et al^[12]^ (n;).

**Figure 1:**
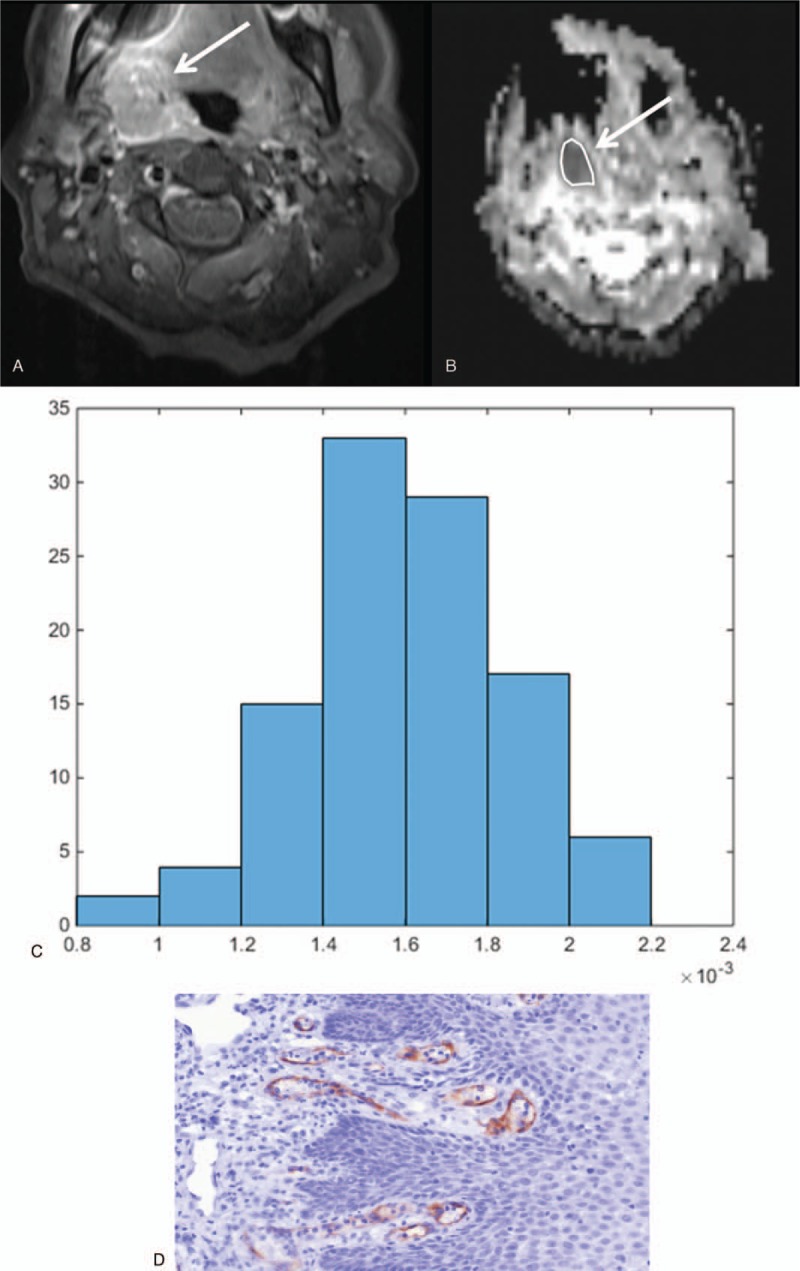
(A) Explanatory patient of our patient sample. Axial slide of a contrast-enhanced t1-weighted image with fat-saturation showing a tonsillar cancer on the right (T2 N2 M0 G3). The arrow points out the lesion. The lesion shows a strong contrast enhancement. (B) Axial slide of the ADC map. The ROI was drawn inside the tumor boundary on every slide with the visible tumor (whole lesion measurement). (C) The corresponding ADC histogram of the tumor. The parameters are as follows: ADCmean 0.96 x 10^–3^ mm^2^/s, ADCmin 0.18 x 10^–3^ mm^2^/s, ADCmax 2.32 x 10^–3^ mm^2^/s, P10 0.54 x 10^–3^ mm^2^/s, P25 0.64 x 10^–3^ mm^2^/s, P75 1.24 x 10^–3^ mm^2^/s, P90 1.41 x 10^–3^ mm^2^/s, ADCmedian 0.90 x 10^–3^ mm^2^/s, ADCmode 0.96 x 10^–3^ mm^2^/s, kurtosis 4.71, skewness 0.88, entropy 2.28. (D) The CD105-stained specimen of the patient. The stained vessel area is 2.23% and the vessel count is 10.

### 2.4 Statistical analysis

For statistical analysis, the SPSS statistical software package was used (SPSS 20; SPSS Inc., Chicago, IL). Collected data were evaluated by means of descriptive statistics (absolute and relative frequencies). Categorical variables were expressed as percentages. *P* values < .05 were taken to indicate statistical significance in all instances. Spearman correlation coefficient was used to analyze the associations between ADC histogram parameters and MVD parameters.

## 3 Results

Table 1 summarizes the investigated ADC histogram parameters and MVD of the patient sample.

**Table 1:**
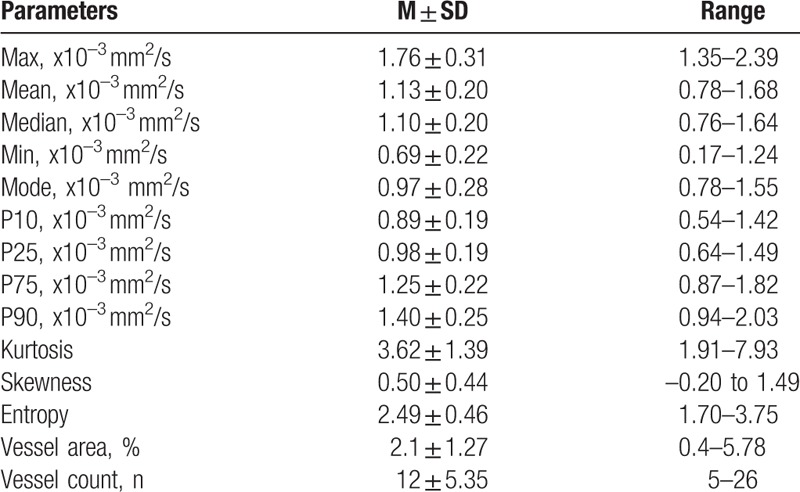
Overview about the investigated imaging and histopathology parameters.

The mean stained vessel was 2.1 ± 1.27% with a range of 0.4% to 5.78% and mean vessel count was 12 ± 5.35 with a range of 5 to 26.

An overview of the Spearman correlation analysis is given in Table 2. There were no statistically significant correlations between ADC histogram parameters and MVD-related parameters.

**Table 2:**
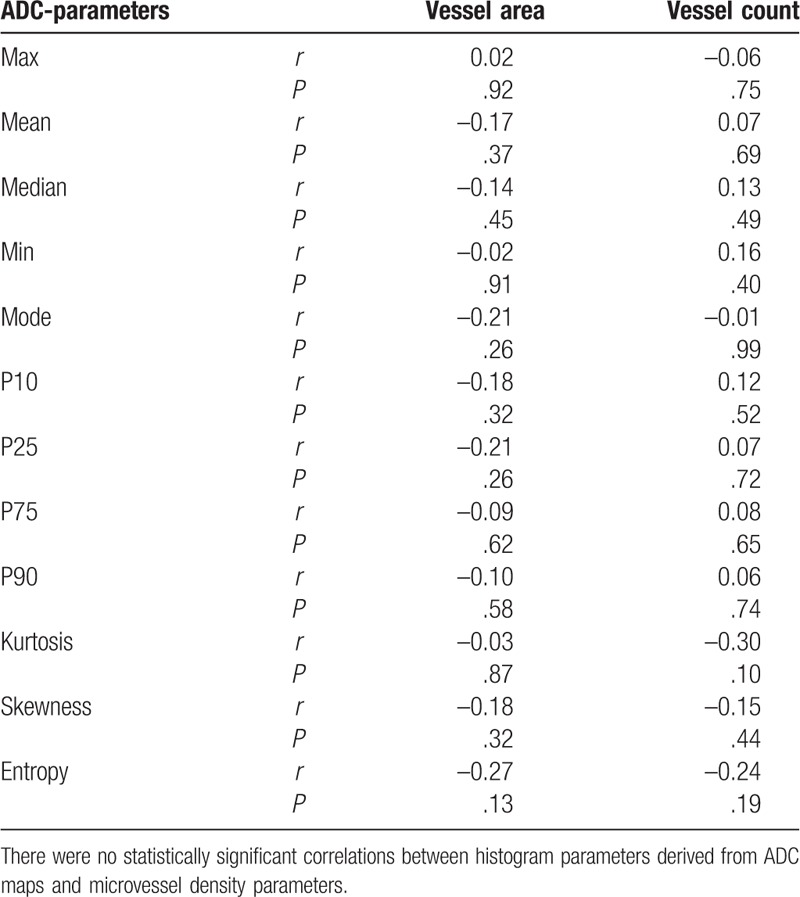
Overview of the correlation analysis.

The calculated correlation coefficients for vessel area ranged from *r* = -0.27, *P* = .13 for entropy to *r* = 0.02, *P* = .92 for ADCmax. Regarding vessel count, the correlation coefficients ranges from *r* = -0.24, *P* = .19 for entropy to *r* = 0.16, *P* = .40 for ADCmin. There were no statistically significant differences after dichotomization according to high and low vessel density in regard of ADC values (*P* > .05).

## 4 Discussion

The present study identified that there are no associations between MVD and whole-lesion histogram parameters derived from DWI in HNSCC. To the best of our knowledge, this finding was not reported previously. Although the study reports negative results, these findings are yet of interest for the following reasons.

DWI is a functional imaging modality that measures the random water motion in tissues and can be quantified by ADC values. In a clinically used DWI protocol, the ADC values are acquired with 2 b-values, a high and a low one, usually 0 and 1000 s/mm^2^, which is similar to the used protocol of the present study. The low portion of the signal intensity up to 200 s/mm^2^ is heavily influenced by perfusion of the capillaries.^[10]^ It is recommended to set the low b-value over 200 s/mm^2^ to measure true diffusion effects.^[20]^

However, to obtain perfusion-related parameters without additional contrast media application, a more advanced DWI technique, namely intravoxel incoherent motion (IVIM), was introduced.^[21]^ It is acquired with multiple b-values with an emphasis in regard of low b-values to measure perfusion parameters. Thereby, several different parameters can be estimated such as perfusion fraction (*f*), pseudo-diffusion (*D*∗), and true diffusion (*D*).^[10]^ According to the literature, these parameters might reflect MVD. For instance, in a gastric cancer mice model, a strong positive correlation was found for *f* and *D*∗ and MVD.^[22]^

However, one major drawback of IVIM is the lack of standardization. For example, various different b-values were used in the published literature. Furthermore, as reported previously, some parameters, such as D∗, might not be reliable enough for clinical translation.^[23,24]^ Therefore, it might be of potential benefit to also predict MVD with a more reliable and clinical routinely used DWI protocol with only 2 b-values. Some previous studies indicated that also ADC values may predict MVD. So far, in cervical cancer, a study used an advanced DWI protocol with a very high b-value of 3000 s/mm^2^ and a low one with 100 s/mm^2^, which observed a strong positive correlation between ADC values and MVD (*r* = 0.940).^[25]^ This finding suggests that also a 2 b-values protocol might be sufficient enough to predict MVD.^[25]^ Moreover, experimental studies observed significant associations between ADC and MVD. For instance, in non-small cell lung cancer tumor models, a complex ADC value with a subtraction technique of high and low b-values was calculated and a weak correlation was found between ADCperfusion values and MVD (*r* = 0.326).^[26]^ However, other studies did not find significant correlations between ADC values and MVD.^[27,28]^

Previous investigations used only mean ADC values to predict MVD. As recently reported, ADC histogram analysis parameters were more sensitive than mean ADC values and can better reflect different histopathological features in several malignancies.^[29–33]^ For example, ADC histogram analysis parameters correlated well with cellularity and proliferation index Ki 67 in HNSCC.^[32]^ Furthermore, it has been shown that ADC histogram values can also predict expression of tumor suppressor gene protein p53 and epidermal growth factor receptor in rectal cancer.^[33]^ We hypothesized that ADC histogram analysis parameters might also reflect MVD. However, the present study could not observe statistically significant correlations between whole-lesion ADC histogram analysis parameters and MVD in HNSCC. This finding indicates that overall ADC values cannot reflect MVD.

There are several limitations of the present study to address. First, although the study is conducted as a prospective study, the data analysis is acquired retrospectively with possible known bias. However, the histopathology and imaging analysis was performed independently and blinded to each other to reduce this limitation. Second, the patient sample is rather small, a limitation similar studies also suffer from. Third, the histopathology was acquired by 1 bioptic sample, which might not be representative for the whole tumor, whereas the MRI was analyzed as a whole-lesion measurement, which might lead to incongruencies. Fourth, the DWI-protocol was a clinically used one with only 2 b-values and therefore no IVIM-related parameters could be calculated. Thus, the study cannot provide information, whether IVIM might be able to reflect MVD in HNSCC.

In conclusion, whole-lesion ADC histogram analysis parameters are not associated with MVD in HNSCC and cannot predict perfusion-related changes in this tumor entity.

## Author contributions

**Conceptualization:** Hans-Jonas Meyer.

**Data curation:** Hans-Jonas Meyer, Anne Kathrin Höhn.

**Formal analysis:** Gordian Hamerla, Anne Kathrin Höhn.

**Investigation:** Hans-Jonas Meyer, Gordian Hamerla, Leonard Leifels, Anne Kathrin Höhn.

**Methodology:** Leonard Leifels.

**Project administration:** Alexey Surov.

**Software:** Gordian Hamerla, Leonard Leifels.

**Supervision:** Hans-Jonas Meyer, Alexey Surov.

**Validation:** Alexey Surov.

**Writing – original draft:** Hans-Jonas Meyer.

**Writing – review & editing:** Alexey Surov.
